# Characteristics of *Planococcus antioxidans* sp. nov., an antioxidant‐producing strain isolated from the desert soil in the Qinghai–Tibetan Plateau

**DOI:** 10.1002/mbo3.1028

**Published:** 2020-03-11

**Authors:** Binglin Zhang, Ruiqi Yang, Gaosen Zhang, Yang Liu, Dongming Zhang, Wei Zhang, Tuo Chen, Guangxiu Liu

**Affiliations:** ^1^ State Key Laboratory of Cryospheric Sciences Northwest Institute of Eco‐Environment and Resources Chinese Academy of Sciences Lanzhou China; ^2^ Key Laboratory of Extreme Environmental Microbial Resources and Engineering Lanzhou China; ^3^ College of Geography and Environmental Engineering Lanzhou City University Lanzhou China; ^4^ Key Laboratory of Desert and Desertification Northwest Institute of Eco‐Environment and Resources Chinese Academy of Sciences Lanzhou China

**Keywords:** antioxidant, *Planococcus antioxidans*, polyphasic taxonomy, Qinghai–Tibetan Plateau

## Abstract

Strain Y74^T^ was an isolate from the sandy soil in the town of Huatugou, Qinghai–Tibet Plateau, China. An analysis of this strain's phenotypic, chemotaxonomic, and genomic characteristics established the relationship of the isolate with the genus *Planococcus*. Strain Y74^T^ was able to grow between 4 and 42°C (with an optimum temperature of 28°C) at pH values of 6–8.5 and in 0%–7% (w/v) NaCl. The dominant quinones were MK‐8 and MK‐7. The polar lipids were diphosphatidylglycerol, phosphatidylethanolamine, phosphatidylglycerol, and an unknown phospholipid. The majority of the fatty acid content was anteiso‐C_15:0_ (28.8%) followed by C_16:1_ ω7c alcohol (20.9%) and iso‐C_14:0_ (13.4%). The 16S rRNA gene sequence similarity analysis demonstrated a stable branch formed by strain Y74^T^ and *Planococcus halotolerans* SCU63^T^ (99.66%). The digital DNA–DNA hybridization between these two strains was 57.2%. The G + C content in the DNA of Y74^T^ was 44.5 mol%. In addition, the morphological, physiological, and chemotaxonomic pattern clearly differentiated the isolates from their known relatives. In conclusion, the strain Y74^T^ (=JCM 32826^T^ = CICC24461^T^) represents a novel member of the genus *Planococcus,* for which the name *Planococcus antioxidans* sp. nov. is proposed. Strain Y74^T^ was found to have potent antioxidant activity via its hydrogen peroxide tolerance and its 1,1‐diphenyl‐2‐picrylhydrazyl (DPPH) radical‐scavenging activity. The DPPH radical‐scavenging activity was determined to be 40.2 ± 0.7%. The genomic analysis indicated that six peroxidases genes, one superoxide dismutase gene, and one dprA (DNA‐protecting protein) are present in the genome of Y74^T^.

## Graphical abstract

In this study, we reported a novel strain isolated from the desert soil in the Qinghai–Tibetan Plateau. According to an analysis of phenotypic, phylogenetic, and chemotaxonomic characteristics, strain Y74T was determined to be a new member within the genus Planococcus. Therefore, it was named as Planococcus antioxidans sp. nov. Y74T. Strain Y74T was found to have potent antioxidant activity via its hydrogen peroxide tolerance and its 1,1‐diphenyl‐2‐picrylhydrazyl (DPPH) radical‐scavenging activity.

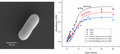

## 1 INTRODUCTION

The accumulation of free radicals in living organisms can lead to many diseases, such as cancer and neurodegenerative diseases (Fischer & Maier, 2015; Lin & Beal, 2006). Thus, it may be possible to reduce and prevent these chronic diseases by decreasing the presence of free radicals and increasing the intake of antioxidants (Bonda et al., 2010; Fischer & Maier, 2015). Microorganisms are an abundant source of bioactive metabolites (Berdy, 2005; Velho‐Pereira, Parvatkar, & Furtado, 2015). Therefore, in order to prevent the toxic effects of free radicals, potent natural antioxidants have been an important target for researchers. Recently, exploring new taxa for new antioxidants has been one of the effective strategies employed in this search.

The Qinghai–Tibet Plateau is the highest plateau in the world, where the average altitude is above 4,500 m (Zhang et al., 2019). Because of the stressful conditions, such as low air temperatures, high UV radiation, and low atmospheric oxygen content, the organisms have had to adapt to survive on this plateau (Zhang et al., 2018; Zhang, Tang, et al., 2016). This environment is a potential source of genetic diversity and is an ideal place to search for antioxidant‐producing microbes (Zhang, Wu, et al., 2016).

The genus *Planococcus* was originally described by Migula (1895). There were 16 valid species in the genus *Planococcus* until recently: *P. citreus* (Migula, 1895), *P. kocurii* (Hao & Komagata, 1985), *P. antarcticus* (Reddy et al., 2002), *P. maritimus* (Yoon, Weiss, Kang, Oh, & Park, 2003), *P. maitriensis* (Alam, Singh, Dube, Reddy, & Shivaji, 2003), *P. rifietoensis* (Romano, Giordano, Lama, Nicolaus, & Gambacorta, 2003), *P. columbae* (Suresh, Mayilraj, Bhattacharya, & Chakrabarti, 2007), *P. donghaensis* (Choi et al., 2007), *P. salinarum* (Yoon, Kang, Lee, Oh, & Oh, 2010), *P. halocryophilus* (Mykytczuk, Wilhelm, & Whyte, 2012), *P. plakortidis* (Kaur et al., 2012), *P. soli* (Luo et al., 2014), *P. faecalis* (Kim, Kang, Yu, Kim, & Lee, 2015), *P. ruber* (Wang et al., 2017), *P. salinus* (Gan, Zhang, Tian, et al., 2018), and *P. halotolerans* (Gan, Zhang, Zhang, et al., 2018). Due to their phenotypic properties, menaquinone profiles, fatty acid composition and G + C content in the DNA, the species *Planococcus mcmeekinii* (Yoon et al., 2001), *Planococcus okeanokoites* (Yoon et al., 2001), *Planococcus alkanoclasticum* (Dai, Wang, Wang, Liu, & Zhou, 2005), *Planococcus psychrophilum* (Dai et al., 2005), and *P. stackebrandtii* (Jung, Kang, Oh, Yoon, & Kim, 2009) were reclassified to genus *Planomicrobium*, and *Planococcus halophilus* was cataloged to the genus *Marinococcus* (Hao, Kocur, & Komagata, 1984; Novitsky & Kushner, 1976). The known features of the genus *Planococcus* are that the species are Gram‐positive, aerobic, non‐spore‐forming and have cell shapes that include cocci, short rods, or rods (Gan, Zhang, Zhang, et al., 2018). The genera *Planococcus* and *Planomicrobium* are close phylogenetic neighbors. Dai et al. (2005) found that the specific difference in the 16S rRNA gene sequence between genus *Planococcus* and *Planomicrobium* at sites 183 and 190 (*E. coli* numbering) was that *Planococcus* contained the signature nucleotides T and A, whereas the *Planomicrobium* species contained C and G (Dai et al., 2005).

According to our research, a new *Planococcus* species strain, Y74^T^, was isolated from the desert soil in the Qinghai–Tibetan Plateau, China. Strain Y74^T^ demonstrated a strong antioxidant activity, which has potential antioxidant applications.

## 2 MATERIALS AND METHODS

### 2.1 Bacteria isolation

The desert soil samples were obtained from the town of Huatugou, Qinghai province, China. Strains Y74^T^ was isolated with modified 216 L agar medium (per liter distilled water: 1.0 g sodium acetate, 10.0 g tryptone, 2.0 g yeast extract, 0.5 g sodium citrate, 0.2 g ammonium nitrate, 0.5 g nutrient broth medium, 20.0 g agar, pH 7.6) and incubated for 7 days at 20°C, after which it was preserved at −80°C in 20% (v/v) glycerol (Wang, Wang, & Shao, 2010).

### 2.2 Genome sequencing and analysis

Genomic DNA was extracted with a bacterial genomic DNA extraction kit (Omega Bio‐tek, Inc.), according to the manufacturer's instructions, and the sequence was determined by the Illumina HiSeq 2000. The reads from the sequencing were assembled de novo using the Velvet 1.2.10 program. The genomes of the type strains that were similar to Y74^T^ were retrieved from GenBank. The average nucleotide identity (ANI) and digital DNA–DNA hybridization (dDDH) were used to assess the degree of similarity of each pair. The ANI was calculated with the JSpeciesWS (Richter, Rossello‐Mora, Glockner, & Peplies, 2016). The ANI could be divided into ANIb and ANIm, depending on the BLASTN (Basic Local Alignment Search Tool) algorithm or the MUMMER ultra‐rapid aligning tool. The dDDH was computed by an online tool, GGDC 2.0: the results of this computation were obtained using the recommended formula 2 (Meier‐Kolthoff, Auch, Klenk, & Goker, 2013). The genome of strain Y74^T^ was annotated using IMG Annotation Pipeline v.5.0.3 (Chen et al., 2019). The G + C content of the DNA of strain Y74^T^ was deduced from the genomic data. Y74^T^ horizontal gene transfer analysis by the method of Bertelli, Laird, and Williams (2017).

### 2.3 Phylogenetic analysis

The closely related type strains of Y74^T^ were obtained by comparing their 16S rRNA gene sequences, retrieved from the genome in the EzTaxon‐e database (Kim et al., 2012). Phylogenetic trees based on the 16S rRNA gene sequences were generated utilizing neighbor joining (Saitou & Nei, 1987), maximum parsimony (Tamura et al., 2011), and maximum likelihood (Felsenstein, 1981) algorithms in MEGA X (Kumar, Stecher, Li, Knyaz, & Tamura, 2018). The sequences were aligned with ClustalW (Larkin et al., 2007). The remaining parameters followed the model of Jukes and Cantor (Jukes & Cantor, 1969), and the bootstrap value was 1,000 re‐samplings (Felsenstein, 1985). A phylogenetic tree based on 25 housekeeping genes nucleotide sequences was generated using neighbor joining algorithms. The parameters were as same as for the phylogenetic tree based on 16S rRNA gene sequences. The sequences of 25 housekeeping genes were obtained from genomes of 19 type strain of genus *Planococcus*, *Planomicrobium,* and 1 outgroup strain (*Lysinibacillus sphaericus* IAM 13420^T^) after annotated using Rapid Annotations using Subsystems Technology (RAST) (Brettin et al., 2015). The sequences of 25 housekeeping genes were concatenated in the following order: CTP synthase, DNA primase, DNA‐directed RNA polymerase beta subunit, LSU ribosomal protein L11p, LSU ribosomal protein L13p, LSU ribosomal protein L16p, LSU ribosomal protein L20p, LSU ribosomal protein L27p, LSU ribosomal protein L3p, LSU ribosomal protein L4p, LSU ribosomal protein L5p, LSU ribosomal protein L6p, phosphoglycerate kinase, ribosome recycling factor, SSU ribosomal protein S10p, SSU ribosomal protein S11p, SSU ribosomal protein S13p, SSU ribosomal protein S2p, SSU ribosomal protein S3p, SSU ribosomal protein S5p, SSU ribosomal protein S9p, tmRNA‐binding protein *SmpB*, transcription termination protein *NusA*, translation elongation factor Ts, and translation initiation factor 3 (Gil, Silva, Pereto, & Moya, 2004).

### 2.4 Morphological and physiological analysis

Cell size and morphology were determined by scanning electron microscopy (JSM‐5600, JEOL) utilizing cells immobilized after gold sputtering for 60 s. For scanning electron microscopy, the strain Y74^T^ was fixed on 4% glutaraldehyde for 8 hr. Subsequently, the cells were dehydrated in an ethanol series (15%, 30%, 50%, 70%, 80%, 90%, and 100%) for 10 min each. Colony color was evaluated on LB (Lysogeny Broth) agar (Oxoid).

Gram staining was tested using the Solarbio Gram staining kit. Growth temperatures (4, 10, 15, 20, 25, 30, 35, 40, 42, and 45°C) and NaCl concentrations (0%–10%, w/v, intervals of 0.5%) were determined on 216L medium. The pH range for growth was examined with strains cultured at 28°C in 216L medium, where the buffering system (KH_2_PO_4_/HCl, KH_2_PO_4_/K_2_HPO_4_, and K_2_HPO_4_/NaOH) was injected to adjust the pH value from 5 to 10 at 0.5 pH unit intervals. Oxidase activity was detected with 1% (w/v) tetramethyl‐p‐phenylenediamine. Starch and gelatin hydrolysis, nitrate reduction, catalase activity, methyl red, and Voges–Proskauer tests were performed according to the description of Kurup and Schmitt (1973). A carbohydrate utilization test was performed as described previously (Zhang et al., 2018). Additional enzyme activities were detected by API ZYM systems.

### 2.5 Chemotaxonomic analysis

For the chemotaxonomic analysis, cells were collected by centrifugation from strains cultured at 28°C in TSB medium (per liter distilled water: 17.0 g tryptone, 3.0 g soy peptone, 2.5 g D‐glucose, 5.0 g sodium chloride, 2.5 g monopotassium phosphate, pH 7.3) for 3 days and then washed twice with distilled water. The cell‐wall peptidoglycan was analyzed by the method of Schleifer and Kandler (1972). The whole‐cell sugars were analyzed by the methods of Lechevalier and Lechevalier (1970). The quinones and the polar lipids were analyzed by the method of Collins et al. and HPLC (Collins, Pirouz, Goodfellow, & Minnikin, 1977; Kroppenstedt, 1982) and by the method of Minnikin et al. (1984), respectively. The methylation, extraction, and analysis of the fatty acids were based on the methods of Sasser (1990) and identified in the TSBA 6.0 database of the Sherlock Microbial identification (MIDI) system (Kämpfer & Kroppenstedt, 1996).

### 2.6 Antioxidant activity analysis

The effect of hydrogen peroxide on the growth of strain Y74^T^ was tested as follows: an inoculum of 100 μl of strain Y74^T^ within the exponential growth phase (OD600 = 0.6) was mixed with 50 ml LB medium containing 0, 1, and 5 mM H_2_O_2_ and then incubated at 30°C for 48 hr. The cell concentration was monitored by spectrophotometer (absorbance at 600 nm). All experiments were performed in triplicate. The growth fitting curves of strain Y74^T^ were drawn with Origin 2018 (logistics nonlinear fitting).

The 1,1‐diphenyl‐2‐picrylhydrazyl (DPPH) radical‐scavenging activity was tested in steps. First, an inoculum of 1 ml of strain Y74^T^ within the exponential growth phase (OD600 = 1.0) was centrifuged at 5,300 *g* for 10 min, after which the supernatant was discarded, and the precipitate was resuspended with 500 μl PBS. This process was repeated three times. The resuspended precipitate was then mixed with 500 μl 0.4 mmol/L DPPH•ethanol (the control group used an equal volume of distilled water), after which the mixture was allowed to react in at low‐light area for 30 min at room temperature and subsequently centrifuged at 5,300 *g* for 10 min. The absorbance of the supernatant was measured with a spectrophotometer at 517 nm. The DPPH free radical‐scavenging rate was calculated as follows: scavenging activity (%) = [1 − (As − Ab)/Ac] × 100%, where Ab is the absorbance of the blank group, Ac is the absorbance of the control group, and As is the absorbance of the sample set.

## 3 RESULTS AND DISCUSSION

### 3.1 Phylogenetic analysis

The entirety of the 16S rRNA gene sequences was extracted from the genome of strain Y74^T^ (1,512 bp, KU601236). The genome of strain Y74^T^ was deposited at DDBJ/EMBL/GenBank with the accession number RCWH00000000.

**Figure 1:**
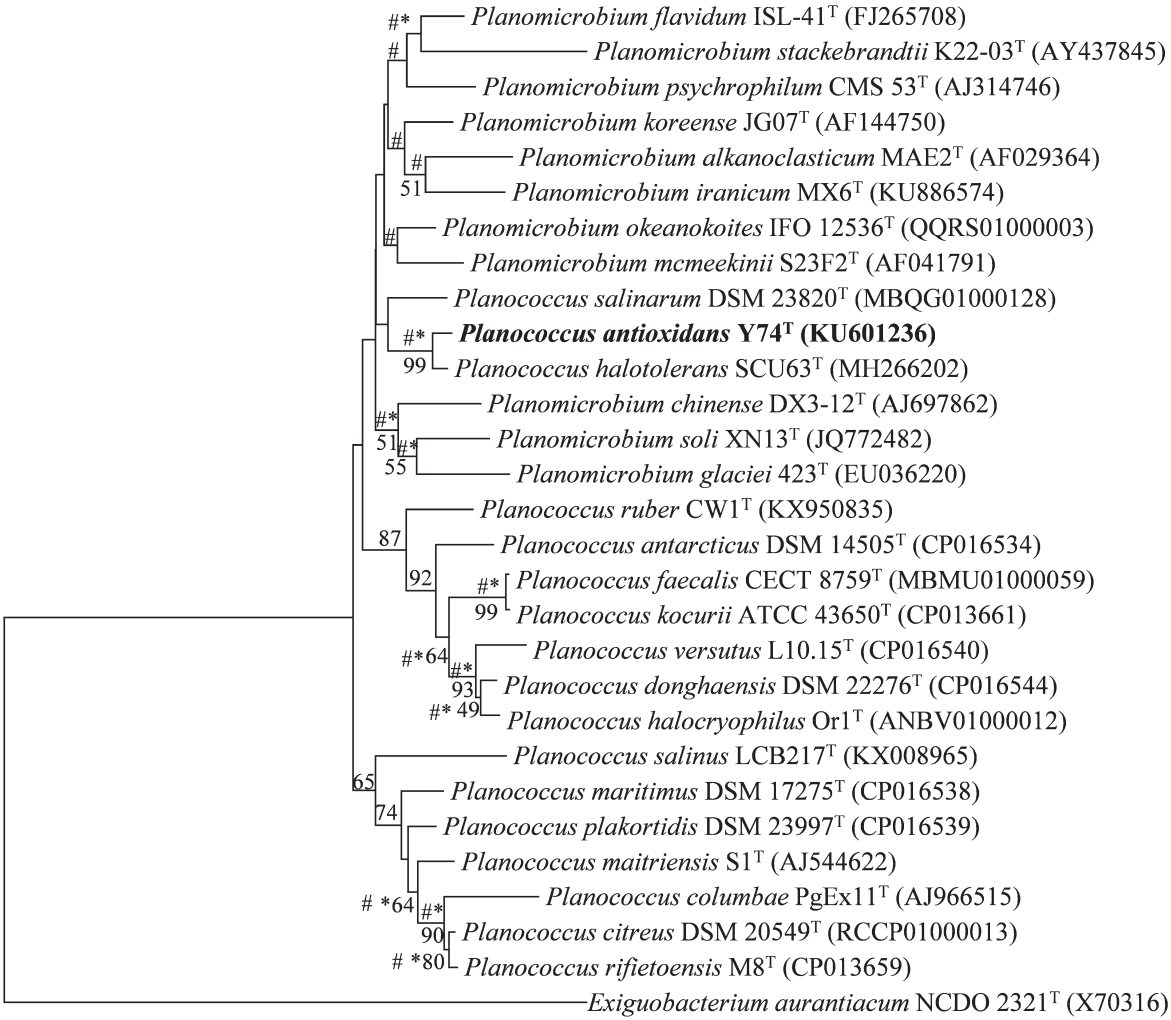
Neighbor joining phylogenetic tree, based on nearly complete 16S rRNA gene sequences, showing the relationships among strain Y74^T^ and their related species. Numbers at nodes are bootstrap values based on 1,000 re‐samplings (only values above 50% are shown). Asterisks and hash marks indicate that the clades were also recovered in the maximum parsimony and maximum likelihood trees

**Figure 2:**
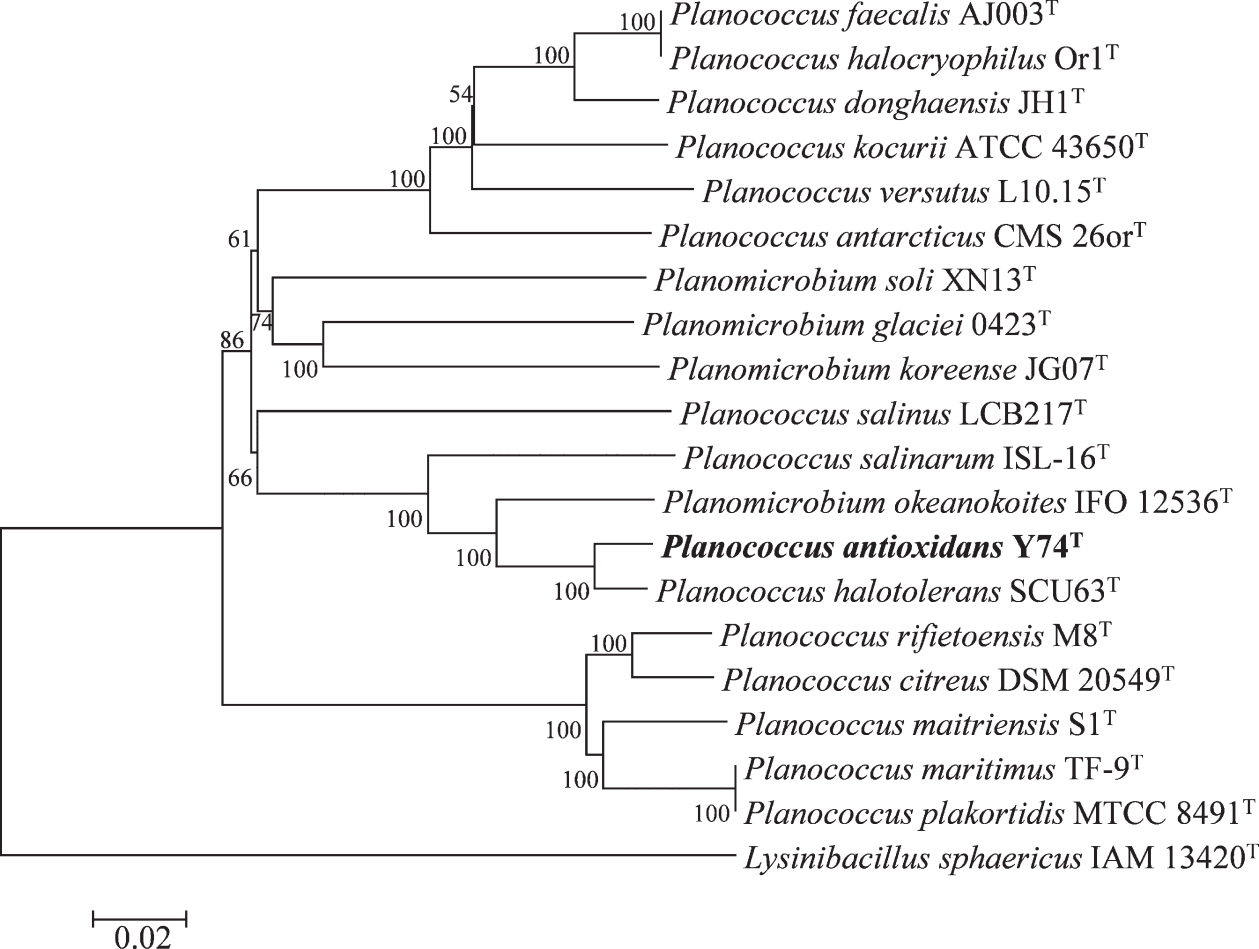
Neighbor joining phylogenetic tree based on 25 concatenated housekeeping genes of strain Y74^T^ and their similar related type strains. Numbers at nodes are bootstrap values based on 1,000 re‐samplings (only values above 50% are shown)

Compared with the EzTaxon database, type strain *Planococcus halotolerans* SCU63^T^ (99.66%), *Planomicrobium okeanokoites* IFO 12536^T^ (98.43%), *Planomicrobium flavidum* ISL‐41^T^ (98.30%), *Planococcus maitriensis* S1^T^ (98.23%), and *Planomicrobium mcmeekinii* S23F2^T^ (98.16%) were found to show high degrees of similarity with strain Y74^T^ (Kim et al., 2012). The three 16S rRNA gene phylogenetic trees indicated that Y74^T^ and *Planococcus halotolerans* SCU63^T^ formed a stable clade (Figure 1). However, many adjacent clades were not stable. Therefore, a more stable phylogenetic tree was constructed, which was based on 25 concatenated housekeeping genes of strain Y74^T^ and their related type strains (Figure 2). According to this phylogenetic tree, strain Y74^T^ should be a member of the genus *Planococcus*. The exact position of *Planomicrobium oekanokoites* IFO12536^T^ could not exactly be resolved within the analysis. The dDDH between strain Y74^T^ and *Planococcus halotolerans* SCU63^T^, *Planomicrobium okeanokoites* IFO 12536^T^, or *Planococcus maitriensis* S1^T^ were 57.2%, 30.5%, and 19.1%, respectively. The ANIb values between strain Y74^T^ and *Planococcus halotolerans* SCU63^T^, *Planomicrobium okeanokoites* IFO 12536^T^, or *Planococcus maitriensis* S1^T^ were 94.15%, 85.43%, and 72.19%, respectively, with ANIm values of 94.66%, 87.45%, and 83.50%, respectively (Table 1). These values were below the species demarcation threshold in prokaryotic species, the generally accepted species boundary for ANI and dDDH values were 95 ~ 96% and 70%, respectively (Chun et al., 2018; Kim, Oh, Park, & Chun, 2014; Meier‐Kolthoff et al., 2013). For all of these reasons, *Planococcus antioxidans* sp. nov. Y74^T^ was designated as a novel species in the genus *Planococcus*.

**Table 1:** The genome comparisons of strain Y74^T^ and the related type species

Species	16S	dDDH	ANIb	ANIm
*Planococcus halotolerans*	99.66	57.2	94.15	94.66
*Planomicrobium okeanokoites*	98.43	30.5	85.43	87.45
*Planococcus maitriensis*	98.23	19.1	72.19	83.50
*Planococcus citreus*	98.10	19.0	72.34	83.74
*Planococcus koreense*	98.10	19.6	73.37	83.96
*Planococcus salinarum*	98.03	24.9	80.89	84.83

### 3.2 Morphological and physiological characteristics

**Figure 3:**
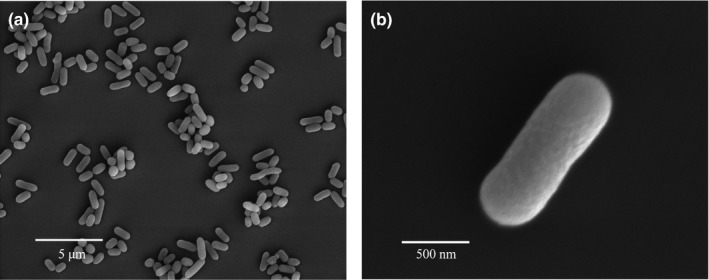
Scanning electron micrograph of strain Y74^T^ (a, b) cultivated on LB medium

Strain Y74^T^ was determined to be Gram‐positive, whose cellular shape was cocci, short rods, or rods, and whose colony color was all white (Figure 3). The growth temperature range of Y74^T^ was 4–42°C (optimum temperature 30°C) with a pH range of 6–8.5 and a NaCl tolerance of up to 7% (w/v) (Table 2). Strain Y74^T^ had a wide range of growth temperatures and a high salt tolerance, which was similar to other closely related type strains of *Planococcus* or *Planomicrobium* (Gan, Zhang, Zhang, et al., 2018; Jung et al., 2009). Strain Y74^T^ could utilize D‐fructose, D‐galactose, D‐glucose, D‐lactose, or D‐maltose as sole carbon sources, weakly utilize D‐cellobiose, D‐lactose, D‐mannitol, D‐mannose, D‐melibiose, D‐raffinose, L‐rhamnose, D‐sorbitol, D‐trehalose, or myo‐inositol, and not utilize starch or sucrose (Table 2). However, *Planococcus halotolerans* SCU63^T^ was able to utilize sucrose but could not utilize D‐sorbitol or melibiose (Gan, Zhang, Zhang, et al., 2018). There were some distinctions with other reference type strains. Strain Y74^T^ showed positivity for catalase and for gelatin hydrolysis and negativity for oxidase, nitrate reduction, methyl red, and the Voges–Proskauer tests (Table 2). The result of the API ZYM test showed that strain Y74^T^ was weakly positive for weak positive for α‐glucosidase, cystine arylamidase, esterase lipase, leucine arylamidase, naphthol‐AS‐BI‐phosphohydrolase, and valine arylamidase.

**Table 2:** Comparison of the characteristics of strain Y74^T^ and the closely related type species

Characteristics	1	2	3	4	5	6	7	8
Cell shape	C, SR, R	C, SR	R	C, SR	C	C, SR	C, SR, R	C,SR,R
Cell length	0.8–3.4	0.4–1.4	1.0–20	2.7–3.3	1–2.0	0.8–1.0	0.5–2.8	0.8–5.0
Cell width	0.8–1.1	0.4–0.6	0.4–0.8	0.4–0.8	1–2.0	0.8–1.0	0.4–0.8	0.4–0.8
Gram stain	Positive	Positive	Positive to variable	Positive to variable	Positive	Positive	Positive to variable	Positive
Colony color	White	Moderate orange	Bright yellow‐bright orange	Light yellow	Orange	Yellow orange	Yellow orange	Pale yellow
Spore formation	−	−	−	−	−	−	−	−
NaCl (%)(w/v)	0–7	15	0–7	0–14	0–12.5	0–10	0–7	0–13
pH	6–8.5	6.5–9.0	7.0^a^	6.0–8.0^b^	6.0–12	5.0–10	5.5–10	6–7.5^b^
Temperature	4–42	0–40	20−37^c^	4–37	0–30	10–45	4–38	4–38
Catalase	+	+	+	+	+	N	+	+
Oxidase	−	+	W	+	−	−	−	+
Nitrate reduction	−	−	−	−	+	+	−	−
Gelatin hydrolysis	W	−	+	+	+	+	+	−
Utilization as carbon sources								
D‐cellobiose	W	+	−	−	−	−	+	−
D‐fructose	+	+	+	+	+	N	−	+
D‐galactose	+	+	−	−	−	N	−	−
D‐glucose	+	+	−	−	+	+	W	−
D‐lactose	W	N	−	−	−	−	+	−
D‐maltose	+	+	−	−	−	N	+	−
D‐mannitol	W	+	−	−	−	−	−	−
D‐mannose	W	+	−	−	−	−	−	−
D‐melibiose	W	−	−	−	+	−	+	−
D‐raffinose	W	N	−	−	+	−	−	−
D‐sorbitol	W	−	−	−	−	N	−	−
D‐trehalose	W	+	−	−	−	N	−	−
L‐rhamnose	W	N	−	−	−	−	−	−
myo‐inositol	W	+	−	−	−	N	−	−
Starch	−	−	−	−	−	−	−	−
Sucrose	−	+	−	−	+	−	−	−
Predominant menaquinone	MK‐8, MK‐7	MK‐8, MK‐7	MK‐8, MK‐7	MK‐8,7	MK‐7,MK‐8	MK‐8	MK‐8, MK‐7, MK‐6	MK‐8, MK‐7
GC content (mol%)	44.5	44.6	46	45.9	39	34.8	47	48.3

Note

Strains: 1, Y74^T^ (data from this study); 2, *Planococcus halotolerans* SCU63^T^ (Gan, Zhang, Zhang, et al., 2018); 3, *Planomicrobium okeanokoites* IFO 12536^T^ (carbon source utilization and enzyme activity test data were from this study, other data were from Nakagawa, 1996); 4, *Planomicrobium flavidum* ISL‐41^T^ (Jung, 2009); 5, *Planococcus maitriensis* S1^T^ (Alam et al., 2003); 6, *Planomicrobium chinense* DX3‐12^T^ (Dai et al., 2005); 7, *Planomicrobium* koreense JG07^T^ (Yoon et al., 2001); 8, *Planococcus salinarum* DSM 23820^T^ (Yoon et al., 2010).

C, cocci; SR, short rods; R, rods; +, positive; −, negative; W, weak positive; N, not determined.

^a^ The range of the growth temperatures was not reported.

^b^ The lower limit was the minimum value that the strain could grow in, and the upper limit was the maximum value of the optimal growth range.

^c^ The optimal growth range.

### 3.3 Chemotaxonomic characteristics

**Figure A1:**
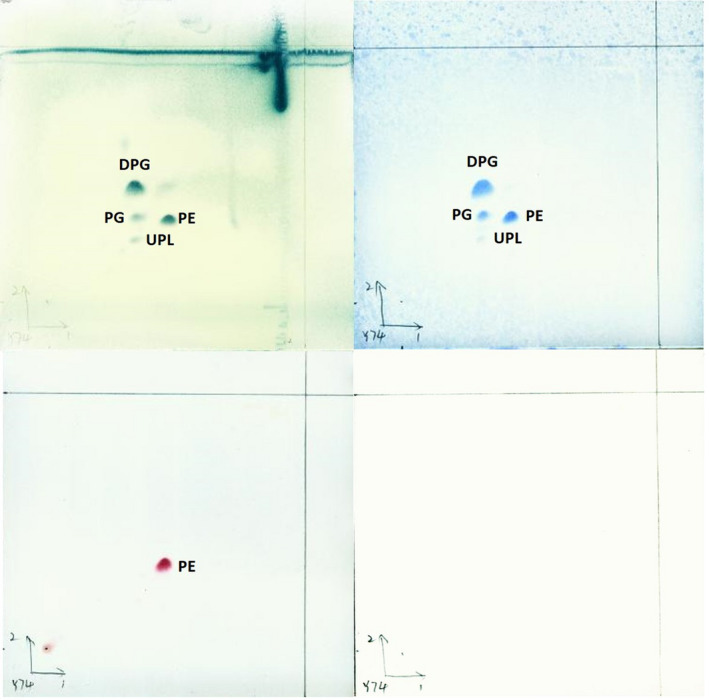
Polar lipid profiles of strain Y74^T^ separated by two‐dimensional thin‐layer chromatography and detected with molybdatophosphoric acid (a), molybdenum blue (b), ninhydrin (c) and alpha‐naphthol (d). The solvent systems used were as following: Direction 1 was chloroform/methyl alcohol/H_2_O (65/25/4, by vol.), Direction 2 was chloroform/ acetic acid/methyl alcohol/H_2_O (80/15/12/4, by vol.). Abbreviations: DPG, diphosphatidylglycerol; PE, phosphatidylethanolamine; PG, phosphatidylglycerol; UPL, unidentified phospholipid

The whole‐cell hydrolysate of Y74^T^ contained ribose. The peptidoglycan type was L‐Lys‐D‐Glu. The dominant quinone compounds of Y74^T^ were MK‐8 (76%) and MK‐7 (24%) (Table 2). Table 2 shows that the predominant isoprenoid quinone compound of the type strains of *Planococcus* and *Planomicrobium* were all MK‐8 and MK‐7. The polar lipids of Y74^T^ were diphosphatidylglycerol, phosphatidylethanolamine, phosphatidylglycerol, and an unknown phospholipid (Figure A1). The major fatty acids of Y74^T^ were anteiso‐C_15:0_ (28.8%), C_16:1_ ω7c alcohol (20.9%), and iso‐C_14:0_ (13.4%) (Table 3). These results conformed to the characteristics of the *Planococcus* genus (Gan, Zhang, Tian, et al., 2018; Kim et al., 2015; Wang et al., 2017).

**Table 3:** Cellular fatty acid composition of strain Y74^T^

Fatty acid	Y74^T^
C_16:0_	1.04
C_18:0_	1.14
iso‐C_14:0_	13.90
iso‐C_15:0_	3.54
iso‐C_16:0_	9.62
iso‐C_17:0_	2.77
anteiso‐C_15:0_	28.81
anteiso‐C_17:0_	2.81
C_16:1_ ω11c	3.02
C_17:0_ 10‐methyl	1.03
C_16:1_ ω7c alcohol	20.88
iso‐C_17:1_ ω10c	3.05
Sum In Feature 4	4.37

Note

The amount of fatty acid was omitted when <1%. Sum In Feature 4: iso‐C_17: 1_ I and/or anteiso‐C_17: 1_ B.

### 3.4 Antioxidant characteristics

**Figure 4:**
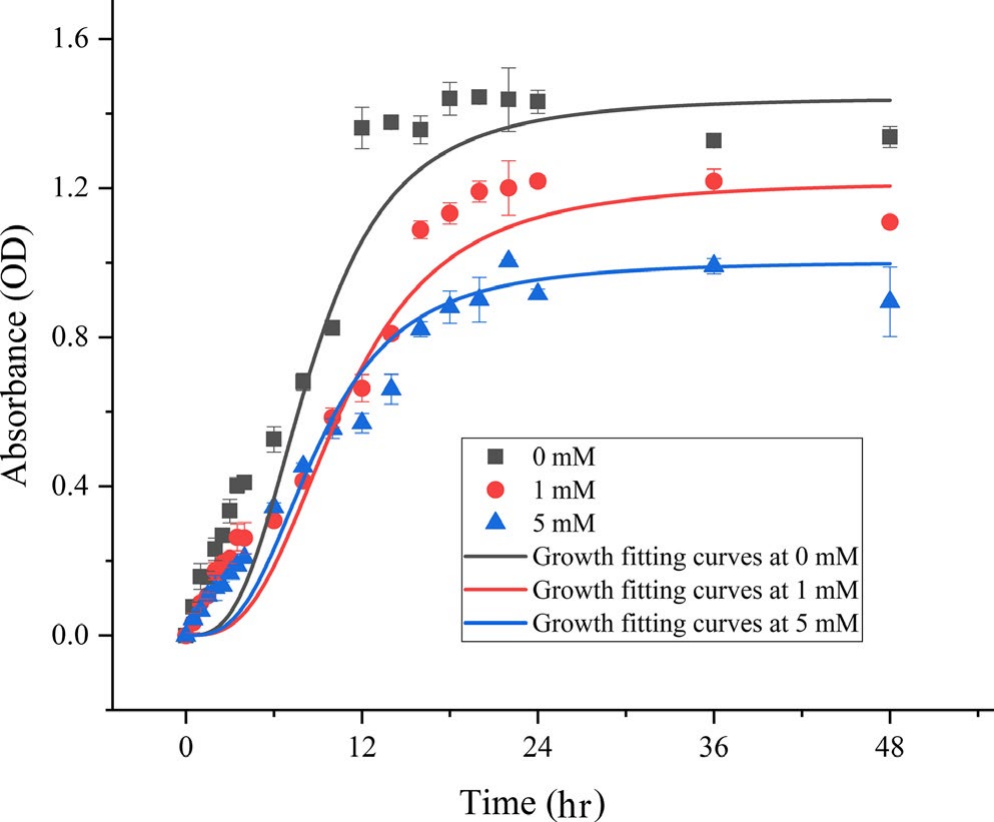
The growth curves of strain Y74^T^ cultivated on LB medium with 0, 1, and 5 mM H_2_O_2_

Strain Y74^T^ showed potent antioxidant activity. The strain grew in LB medium supplemented with 1 or 5 mM hydrogen peroxide. The growth curves of Y74^T^ in LB medium supplemented with 0, 1, and 5 mM hydrogen peroxide were similar. Strain Y74^T^ reached a stable growth phase after approximately 18 hr under three conditions. However, the cell concentrations were significantly different, with the highest cell concentration observed in the LB medium with 0 mM hydrogen peroxide and the lowest cell concentration seen in the LB medium with 5 mM hydrogen peroxide (Figure 4). The DPPH radical‐scavenging activity was 40.2 ± 0.7%. There were many studies on antioxidants of genus *Lactobacillus,* which could produce antioxidants and had extensive application (Das & Goyal, 2015; Le & Cao, 2010; Lee et al., 2010). The strain Y74^T^ had the significant resistant ability to hydrogen peroxide than many species of *Lactobacillus*, such as *Lactobacillus plantarum* DM5, *Lactobacillus plantarum* NRRL B‐4496, and *Lactobacillus* acidophilus NRRL B‐4495 (Das & Goyal, 2015). Simultaneously, the radical‐scavenging ability of DPPH of strain Y74^T^ was higher than *Lactobacillus plantarum* NRRL B‐4496 but slightly lower than *Lactobacillus acidophilus* NRRL B‐4495 (Das & Goyal, 2015; Kaizu, Sasaki, Nakajima, & Suzuki, 1993). This indicates that the antioxidant activity of strain Y74^T^ had reached a practical level. The species of *Planococcus* genus were always isolated from some extreme environments, such as the Arctic permafrost or the Antarctic (Mykytczuk et al., 2012; Reddy et al., 2002). Therefore, these strains have strong stress‐resistant abilities generally. Previous research found a new antioxidant in *Planococcus* (Shindo & Misawa, 2014). The species of *Planococcus* genus had the potential for antioxidant applications, but few studies were emphasized on this property.

### 3.5 Genome properties

The draft genome of strain Y74^T^ was 3,672,033 bp (Table 4). The G + C content of the DNA of Y74^T^ was 44.5 mol%. A total of 3,831 genes were detected in strain Y74^T^, 3,668 of which were protein‐coding genes. There were 967 protein‐coding genes containing enzymes. The number of protein‐coding genes with a function prediction was 2,913. The number of protein‐coding genes with COGs was 3,006, which accounted for 78.47% of all genes. The genome of strain Y74^T^ contained 106 RNA genes, including 67 tRNA genes, 10 5S rRNA genes, 7 16S rRNA genes, and 12 23S rRNA genes (Table 4). The copy number of the rRNA operons in prokaryotic organisms is generally thought to be related to growth rates (Klappenbach, Dunbar, & Schmidt, 2000). It could be suggested that strain Y74^T^ could grow rapidly at lower temperatures. Multiple copy numbers of key genes could increase the radiation resistance of the bacteria as well (Slade & Radman, 2011). There are six peroxidase genes in the genome of Y74^T^ (Table A1), including two glutathione peroxidases, one catalase family peroxidase, one heme‐dependent peroxidase, one thioredoxin‐dependent thiol peroxidase, and one thiol peroxidase. Peroxidases are enzymes that catalyze the oxidation of substrates by hydrogen peroxide as an electron acceptor (Welinder, 1992). This property may explain why the strain can grow in a medium containing hydrogen peroxide. Genes with antioxidant abilities, DNA‐protecting protein (DprA), and superoxide dismutase were also found in the genome of Y74^T^.

**Table 4:** Summary of *Planococcus antioxidans* Y74^T^

Feature	Genome	
	Value	% of total
Size (bp)	3,672,033	100
Coding region (bp)	3,150,934	85.81
Total genes	3,831	100
RNA genes	106	2.77
tRNA	67	1.75
5S rRNA	10	0.26
16S rRNA	7	0.18
23S rRNA	12	0.31
Other RNA genes	10	0.26
Protein‐coding genes	3,668	95.75
Protein‐coding genes with function prediction	2,913	76.04
Protein‐coding genes with enzymes	967	25.24
Protein‐coding genes coding signal peptides	178	4.65
Protein‐coding genes coding transmembrane proteins	987	25.76
Protein‐coding genes with COGs	3,006	78.47

**Figure 5:**
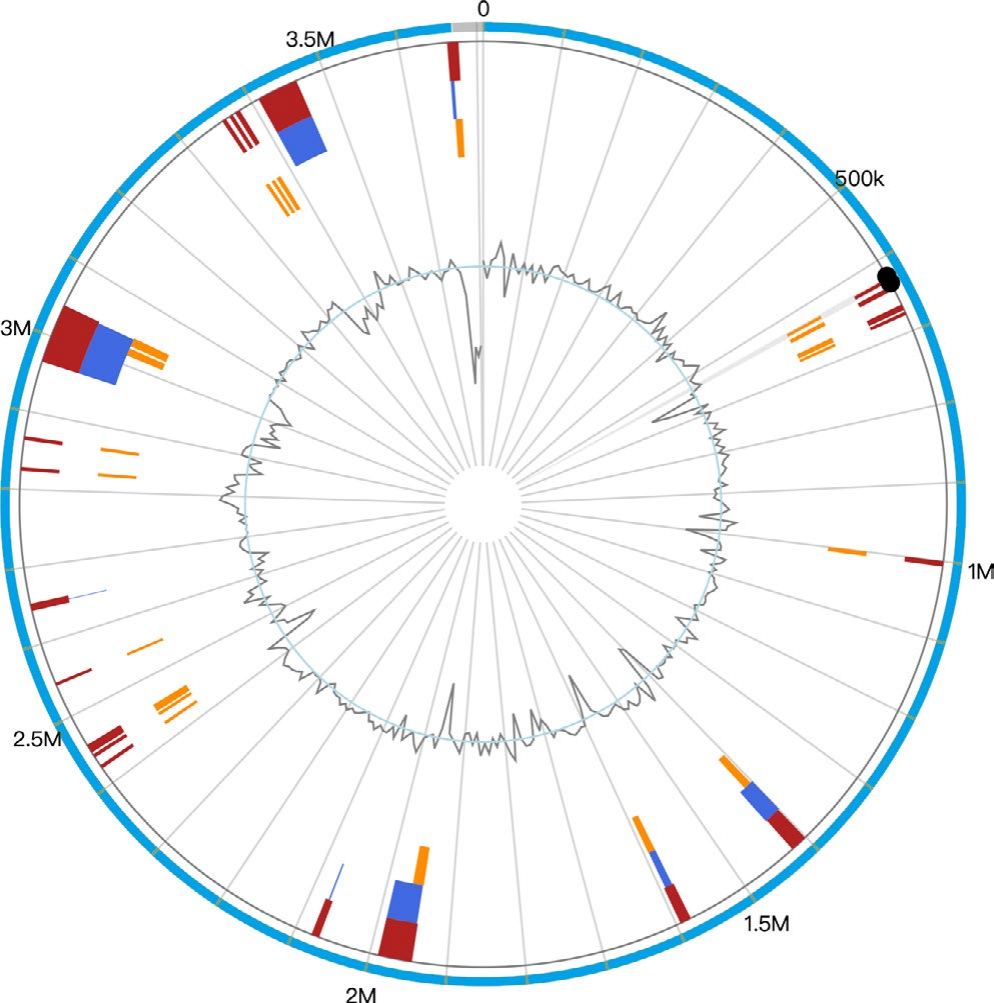
Characteristics and position of the predicted genomic islands found in the draft genome sequence of strain Y74^T^. The genomic islands show that several horizontal gene transfer events have occurred in strain Y74^T^. The red in the circles represents the prediction from integrating three different methods (IslandPath‐DIMOB, SIGI‐HMM, and IslandPick); the orange represents the prediction result using IslandPath‐DIMOB; the dark blue represents the prediction result using SIGI‐HMM

In addition to the core proteins and other orthologs presenting in the organism, some nonortholog proteins were found in the strain of Y74^T^. Therefore, the gene cluster of strain Y74^T^ was analyzed by the method of Bertelli et al. (2017). The results showed that multiple horizontal gene transfer events were found in the Y74^T^ genome. There were 28 gene islands be found in the genome, which contained 395 genes ranging from 4,000 to 700,000 bp, including 162 unclear functional genes of them were annotated as hypothetical proteins; 16 genes were predicted to be recombinase and phage‐associated proteins (Figure 5, Table A2). Based on the gene function analysis on gene island, most of the genes were involved in metabolism, signal transduction, and DNA repair.

## 4 CONCLUSIONS

According to an analysis of phenotypic, phylogenetic, and chemotaxonomic characteristics, strain Y74^T^ was determined to be a new member within the genus *Planococcus*. Therefore, it was named as *Planococcus antioxidans* sp. nov. Y74^T^. Strain Y74^T^ was found to have potent antioxidant activity via its hydrogen peroxide tolerance and its DPPH radical‐scavenging activity.

### 4.1 Description of *Planococcus antioxidans* sp. nov

*Planococcus antioxidans* (an.ti.o'xi.dans. gr. pref. anti, against; N.L. v. oxidare, to oxidize; N.L. part. adj. antioxidans, referring to the characteristic of this strain).

The colonies on LB agar (Oxoid) are circular, smooth, and white. The cells are aerobic, Gram‐stain‐positive, non‐spore‐forming, and occur as cocci, short rods, or rods (0.8–1.1 × 0.8–3.4 μm). Growth occurs between 4 and 42°C (optimum temperature 28°C), pH values of 6–8.5 and 0%–7% (w/v) NaCl. Strain Y74^T^ utilizes D‐fructose, D‐galactose, D‐glucose, D‐lactose, or D‐maltose as sole carbon sources, weakly utilizes D‐cellobiose, D‐lactose, D‐mannitol, D‐mannose, D‐melibiose, D‐raffinose, L‐rhamnose, D‐sorbitol, D‐trehalose, or myo‐inositol, and does not utilize starch or sucrose. Strain Y74^T^ shows positively for catalase and for gelatin hydrolysis, and negativity for oxidase, nitrate reduction, methyl red, and the Voges–Proskauer tests. In assays with the API ZYM system, strain Y74^T^ is weakly positive for α‐glucosidase, cystine arylamidase, esterase lipase, leucine arylamidase, naphthol‐AS‐BI‐phosphohydrolase, and valine arylamidase, and negative for α‐chymotrypsin, α‐fucosidase, α‐galactosidase, α‐mannosidase, acid phosphatase, alkaline phosphatase, β‐galactosidase, β‐glucosidase, β‐glucuronidase, esterase, lipase, N‐acetyl‐β‐glucosaminidase, and trypsin. The cell wall contains ribose. The peptidoglycan type was L‐Lys‐D‐Glu. The dominant quinones are MK‐8 and MK‐7. The polar lipids are diphosphatidylglycerol, phosphatidylethanolamine, phosphatidylglycerol, and an unknown phospholipid. The majority of the fatty acid is anteiso‐C_15:0_ (28.8%), followed by C_16:1_ ω7c alcohol (20.9%) and iso‐C_14:0_ (13.4%).

The type strain, Y74^T^ (=JCM 32826^T^ = CICC24461^T^), was isolated from the sandy soil in the town of Huatugou, Qinghai province, China. The G + C content of the DNA of strain Y74^T^ is 44.5 mol%.

## CONFLICT OF INTEREST

None declared.

## AUTHOR CONTRIBUTION

Binglin Zhang: Conceptualization‐Equal, Software‐Equal, Writing‐review & editing‐Equal; Ruiqi Yang: Investigation‐Equal, Resources‐Equal; Gaosen Zhang: Methodology‐Equal, Resources‐Equal; Yang Liu: Formal analysis‐Equal; Dongming Zhang: Formal analysis‐Equal, Software‐Equal; Wei Zhang: Methodology‐Equal; Tuo Chen: Conceptualization‐Equal, Writing‐review & editing‐Equal; Guangxiu Liu: Conceptualization‐Equal, Writing‐review & editing‐Equal.

## ETHICS STATEMENT

None required.

## Data Availability

All data are provided in full in the results section of this paper apart from the DNA sequences. The 16S rRNA gene sequence of strain Y74^T^ is available in the GenBank database under accession number KU601236. The genome of strain Y74^T^ is deposited in GenBank with accession number RCWH00000000. The type strain Y74^T^ is deposited in the Japan Collection of Microorganisms (https://www.jcm.riken.jp/cgi‐bin/jcm/jcm_number?JCM=32826) and the China Center of Industrial Culture Collection (http://sales.china‐cicc.org/category.php?id=1&sh=jd&keywords=24461).

## Appendix A

**Table A1:** Antioxidant related genes of strain Y74^T^

Gene	Protein	Length	DNA sequence
cds_RLQ92685	DNA‐protecting protein *DprA*	906	ATGGATTCATTGTTTGAACAAAGACTGATGGCATTGCATTATGTATACCCTCAACCACTCAACCGCATAAAACGGCTGATGATTGACGATTCGAATCTTGAACATTTGGAAAGCAGGCCAGCCTGGGAAATCAGCCAATTACTCGGCATAAAGCCGGAAGCGGCGATATCACTGAAGGATGCTTATAGAAAATCACTGAACAACCCCTATTCTGAAACTTATGAAAAACATAAGATAATCCCCATATCTTATAACCATCCCAATTATCCACAAAGTCTATTTCATCTGATCGACCCACCTGTAATTCTTTACGCCAAAGGAAAAATAGAGTACTTGCTGAATGAAGATCGGATAGCTGTGATAGGTGCCCGTAAGGCTTCTGTTTATTCACAGAAAGCTATGGATCTTATACTTCCTGATCTTGTTGCAGCGGGCTTTATCGTGGTAAGCGGCTTGGCAAAAGGGGCAGATGCAATGGCTCACCGGACAGCAATCGATTGCGGCGGCAAAACGATTGCTGTTACCGGCAGCGGCTTTTTGCATCCGTATCCGAAAGAGAATGATGAATTGAATATTATAATAGAAGAAACTCAACTCGCAATTACAGAATATCCGCCATATATGCAGCCGAAACGCTGGAATTTCCCTATGCGGAACCGCATTATAAGCGGCTTGGCAAAAGGGGTACTGGTAACGGAAGCGGAAGTGAAAAGCGGCACGCTCAGCACGATTGAACATGCCCTGGAACACGGCAAGGATATTTTTGCGGTACCAGGGAGTATCTGTTCACCTCTGTCAGCCGGGCCGCATAAACTGATTTTTGAAGGTGCAAAACCGGTCTGGAATGGGCTTCAAGTGCTGGAGGAATACCGTGAAATTAGGGCTTTAAATAAGTCGATAAAATGA
cds_RLQ92091	Superoxide dismutase	609	ATGGCTTATGAATTACCGGAACTACCTTACGCGTATGACGCACTGGAACCACACATCGACAAAGAGACGATGAATATTCACCACACAAAACACCATAATACTTACGTAACCAATGTTAACGCTGCCCTGGAAGGCCACGAAGATCTTTCTTCAAAATCTGTAGAAGAACTGATTTCTGACTTGAACGCTGTGCCTGAAGATATTCGTACAGCTGTACGCAATAACGGCGGTGGACACGCAAACCACTCATTATTCTGGCAATTATTGACTCCAAACGGCACTGGCGCTCCATCAGGTGCACTTGCGGAAGCAATCGACAGCAAGTTCGGCAGCTTTGACGAATTCAAAACGAAATTCGAAGCAGCCGGTAAAACACGCTTCGGTTCAGGCTGGGCTTGGCTTGTTGTATCTAATGGTGAATTGGAAGTAACTTCAACTGCCAACCAGGATTCTCCATTGATGGACGGCAAAACGCCAATTCTTGGAGTAGACGTTTGGGAGCATGCTTACTACTTGAAATACCAGAACAAACGCCCTGACTATTTGGCTGCTTTCTGGAACGTAGTAAACTGGGACGAAGTTTCAAAACGCTATGAAGCTGCAAAATAA
cds_RLQ91554	Glutathione peroxidase	477	ATGAGTATTTATGAATTTTCGGCCAGAAAGTCCGATGGCAGCATTTATCCGTTAAGTGAATACGAAGGGAAAACGATGTTGATTGTCAATACTGCTACGAAATGCGGGTTGCGTGATCAATTCGATGGACTGGAGAAGTTGTACCAGAAGTATGAAGATGACGGACTTGTCGTCCTTGGCTTTCCTTCCGATCAGTTCGGGCAGGAACTTGATGGGGCGAAAGAAGCGGAGGAATCCTGCCGGATGACTTACGGAGTTTCGTTCCCAATGCATGACCTGGTCAAAGTTAACGGAAAAAATGCCGATCCTCTTTTTAAATATCTAACTGAAAACAGCAAGGGAGTCCTTGGCAGCAGCATCAAGTGGAATTTCACAAAATTTCTCATCAACAAAGAGGGAAAGCTGGTTGCACGGTTTTCGCCAAAAGATAAACCTGAAAAATTTGAAGAAGAAATTAAACAATACTTAACGAACTGA
cds_RLQ90872	Catalase family peroxidase	927	ATGGCGAAGGAAAAGCTTGCGGAAACCGCAGTCAATAAAATCGAGAAAGTGTTTGGGGAACATAAAACTTATAGACGTGCGCATTCAAGAGGAACGGGATATGAAGCCCTATTCACAGCAAACGGCGAAGGGCAGAATTGGACCGTCGCGCAGCATCTCCGGGAAGGGACGACCAAAGCTGTGGTGCGATTTTCGCACAGTTCTCCAGATCCATTTTGGACGGATAATTTATCACCGGTAAAAGGAATGGCTGTGCAATTCCAGTTGCCGGATGGCCAAGTGATGAACAGTGTCGGCGTAACTTCCCCGATATTCTTTTCGCGCACTCCGGAAGTGTTTACGGAAATGCTGGATATCGCGAAATCGTTTAAAAAGGGCAAGCCCCGGCTGCGGGATCTCATCAAATTGTTCATCAAATATCCCGAAAGCCGAGCAGCAATCCGCATCATCCGGAAAATGCAGAGTCCGGCTAGTTTCGCGACCGGTCTCTACCATTCCATTCACGCATTTTACCTGGTTAACGGTACTGGCCAGCGCGTACCCGTCAAATTCCAGTGGCATCCGGAAGCGGGCGTGGAGTCGTTGAATCCGGTGGAGGCTGCGTCAGTGAAAAAAGGAGATTTTGAGGAAGAGCTTGAAGAACGCGTCTTGAGCGGAGAGACGGCTTTCCGTCTGATGGCAGTGATTGGGGATGCGGATGACCCTGTAGATGACCCGACGAAAGACTGGGCTAAAGATAGAAAGAAAGTCGAATTGGGGCGCCTGGTGCTGAAAGAACAGACTGACGAAGCGGAAGGGTTGCTGTATGATCCAACCATCCTGGCAGAAGGCGTCGAATGCACGGATGATCCCATCCTGCATTTCCGCAACCCGGCATATGCTATTTCTTATATGCGGAGAGAAGGGGAGAAGCAAAAAGAAAGTTGA
cds_RLQ90525	Heme‐dependent peroxidase	750	ATGAATGAAGCAGCAATCACTTTAGACGGCTGGTACGTCCTCCACGATTTCCGTTCGATGGACTGGGTATCATGGAAAATGCTTGAAGACGAAGAACGCCAATTCGCAATCGACGAATATCAGGCATTCATGGACAAAGTAAACCAGGCCGATGAAAATAAAACCGGTGCACACGCATTGTATTCAATTATTGGCCAAAAAGCTGACTTGATGCTGATGCTATTGCGCGAAACTATGGACGAATTGCGTGAACTTGAAACGGAATACAATAAGCTGACATTGGTCGCTTACACGGTTCCGACTTACTCTTACGTATCTGTAGTGGAACTTTCCAACTATCTTGCAGGTAAATCAGAAGAAGATCCATACCAGAACCCGCATGTCCGCGCGCGTCTGTATCCGGAGCTTCAGCGTTCGCAGTACATCTGCTTCTACCCGATGGACAAGCGCCGCGACGGCAACGACAACTGGTACATGCTGCCGATGGACGAGCGCAAAGATTTGATGCTGTCACACGGCAAAATCGGCCGCAGCTACGCAGGCAAAGTAAAACAGATCATTTCCGGCTCTGTCGGCTTTGATGATTACGAATGGGGCGTAACCTTGTTCGCAGATGACGTTCTGCAGTTTAAAAAACTGATCTATGAAATGCGTTTTGACGAAGTCAGCGCGCGTTACGCTGAATTCGGTTCGTTCTACGTCGGCACTCGCCTTGATAAAGAAAGAATCGTTAAGTTTTTGGAAGTTTAA
cds_RLQ82443	Thioredoxin‐dependent thiol peroxidase	477	TTGACAACATTAGAAGGTTTGCATGCACCGGATTTTACATTGAAAAATGAAAACGGCGAAACAGTTTCTTTGGAGGATTTTGCCGGCAAAAAATACGTAGTGCTTTATTTTTACCCGAAAGATATGACACCGGGCTGCACTACACAGGCCTGCGATTTCCGGGATGCAGAGAAGGATTTTTCCGAATTGGGAGCAGTCATTCTTGGCGTTAGCGCAGACTCTGAAAAACAGCACAGTAAATTTATCAGCAAACACGGTTTGCCATTCTCTTTATTGGTTGACGAAGATCATAAAGTTTCTGAGGCATACGGTGTGTGGGTGGAGAAGAAGATGTACGGAAAAGAATTTATGGGGATTGAACGCTCTACATTTTTAATCGACCCAACCGGGACTGTCGTAAAAGAATGGCGAAAAGTCAAAGTGAAAGACCATATCCAGGAAGTCCTTGAAACGGTCAAAGAGCTCAGCCAAGCATAG
cds_RLQ81587	Glutathione peroxidase	477	ATGGTTTCGGTTTATGATTACAAAGTTAAAAATTTGCAGGGAGAAATGGAATCACTTGAAAAGTTTAAAGGGAATGCGCTGGTAATAGTCAATACAGCAAGCAAATGTGGATTGACTCCTCAATTTGAAGACCTTCAAAAACTCTATGAAAAGTATTCCAGTAAAGATTTTCAAATCCTCGGTTTTCCAAGTTCTCAATTTAATAATCAGGAATTTGAAAATCAGGAAGAAACGATGGAATTCTGCCAGATGAATTATGGTGTAACATTTCCTATGTTTGCAAAAACAGATGTCAAAGGAGCAGATGCAGCTCCACTATTTACCTATCTGACTTCAAAGCATGAAAATCTAGAGGCTGAAGAAATTGCATGGAACTTTGCGAAGTTTTTAATAGATAAAGAAGGACATGTCATTAAAAGATATTCCCCTCGATCTTCGCCACTGGAAATTGAAGAAGACCTGAAGACTATTCTATAA
cds_RLQ91872	Thiol peroxidase	507	ATGGTACAAGTTACATTTCATGAAAATCCTGTTACCTTACCAAACAAAGAAGTCAAAGTTGGAGACCAAATCCCGAATTTCACAGTACTTGACAATGACTTGAACCCTGTAACTGCACAGGATACAGCAGGTAAAGTCAGATTATTCACAGTGTCACCATCCTTGGATACAAGTGTTTGTTCAGATCAGGCGAAACGTTTCAGCGAAGAAGCTTCATCAATGGGAGACGAAGTTGCAATCTACTATGTAACCTGCGATTTGCCTTTCGCACAAAAGCGTTGGGTTGAAGTTAATGAAGCCAAAAATCTCACTACTCTTTCCGATCACCGTGATCTTTCTTTCGGTGAAGCGTTCGGAGTAACGATGCAGGAACTGCGTTTGCTGGCCCGTTCCATATTTGTAGTGGATGAAAACGATAAAGTGACTTATGTGGAATATGTTCCTGAGGGAACGAATCATCCGAATTACGATAAAGCGATTGAAGCAGTAAAAGAACTGACAAAATAA

**Table A2:** Characteristics of the genomic islands found in the genome of strain Y74^T^

GI	Length	Total no. of gene	hypothetical proteins	Integrase/ phage	Predicted function
1	4,681	6	2	0	Metabolism/ Transcription factors
2	6,367	8	2	0	
3	7,602	6	3	0	General function prediction only/ Metabolism
4	4,374	3	2	0	Metabolism
5	5,080	6	4	0	
6	6,976	5	3	0	Replication and repair
7	21,818	19	7	1	Genetic information processing /Transcription factors/Cell processing/ Nucleotide metabolism
8	12,222	10	6	1	Transcription factors/DNA repair
9	14,623	20	14	0	Secretion system/DNA repair
10	11,232	14	11	0	
11	16,787	17	4	2	Transporters/Carbohydrate metabolism/ Transferases/Two‐component system
12	42,958	29	4	2	Two‐component system/ Metabolism/Transcription factors
13	10,266	7	1	0	Metabolism/DNA repair
14	4,777	7	1	0	
15	4,674	5	3	0	
16	11,402	9	4	0	
17	9,706	11	4	1	Metabolism/Transcription factors/ Transferases /Signal transduction
18	53,470	60	25	0	
19	4,656	6	0	0	Metabolism/Membrane transport
20	75,201	74	30	2	Secretion system/Metabolism/Transcription factors/Membrane transport
21	11,374	13	10	1	Genetic information processing
22	13,041	16	6	1	Metabolism/Transcription factors
23	5,465	9	4	0	Transcription factors/Genetic information processing
24	5,113	6	1	0	Transcription factors/Transferases
25	6,724	10	3	0	
26	4,021	6	3	0	
27	6,818	8	4	2	Transcription factors
28	10,911	5	1	3	
Total	392,339	395	162	16	
